# Children’s oral health-related quality of life and associated factors: Mid-term changes after dental treatment under general anesthesia

**DOI:** 10.4317/jced.51906

**Published:** 2015-02-01

**Authors:** Ziad D. Baghdadi

**Affiliations:** 1 DDS, PD, MS, MPH, PhD. Department of Preventive Dentistry, Riyadh Colleges of Dentistry and Pharmacy, Riyadh, Saudi Arabia; 2 DDS, PD, MS, MPH, PhD. Department of Community Health and Epidemiology, College of Medicine, University of Saskatchewan, Saskatoon, SK, Canada S7N 5E5

## Abstract

Objectives: This study aimed to document the mid-term effects of comprehensive dental treatment under general anesthesia (DTGA) on parent-assessed children’s oral health-related quality of life (COHRQoL). A second aim was to examine some epidemiological factors associated with COHRQoL and treatment outcome. Study Design: A pretest-posttest design was followed in which parents were surveyed using the Child Oral Health Quality of Life Questionnaire before and 6-9 months after their children (age ranges 3-10 years) underwent DTGA. Some clinical conditions and epidemiological factors were examined to assess their association with COHRQoL and changes resulting from treatment. Results: The clinical sample consisted of 80 children-parent dyads. The effect sizes of change following DTGA were large for both the child impact section and family impact section of the COHRQoL. COHRQoL scores after treatment were comparable or lower than those of a cross-matched group of children with no complaints related to oral health. Child’s age, pain and number of teeth with pulpal involvement showed significant association with both pretreatment scores and change scores. Conclusions: Children’s OHRQoL improved after DTGA as assessed by parents 6-9 months postoperatively. Child’s age, pain and number of pulpally-involved teeth can be used as predictors for COHRQoL and change scores.

** Key words:**Quality of life, children, oral health, reliability.

## Introduction

The definition of oral health as the standard of oral health and related tissue health that enables individuals to eat, speak, and socialize without active disease, discomfort, or embarrassment, and that contributes to general wellbeing is in line with the World Health Organization’s general definition of health as a state of complete physical, mental and social wellbeing and not merely the absence of disease or infirmity (1,2). The recent interest in oral health-related quality of life [OHRQoL] reflects scientists’ recognition of the interaction between oral health conditions and social factors, contextual factors, and the rest of the body [i.e., health as defined by the WHO]. Though preventable, dental caries in children is a public health concern, which can impair children’s ability to eat, interact with others, or attend school (3). Severe oral health problems can cause children needless pain and suffering along with devastating complications undermining an individual’s wellbeing concomitant with financial and social costs, diminishing quality of life and burdening a society. OHRQoL is being recently used to measure efficacious of treatment to improve care. This is congruent with patient-centered care, provides evidence that costs and resources associated with some treatment protocols are worth the expense, and assists patients [and families] in treatment decision-making (4). Dental treatment under general anesthesia [DTGA] in children is one of treatment protocols that has captured some attention by examining changes in OHRQoL following DTGA among young children with severe dental caries. All the studies published so far have documented short-term [1 – 8 weeks] changes after DTGA (2,4-9). These studies imply that OHRQoL does improve after DTGA; however, Klaassen *et al.* (6) found that the improvement of the quality of life was relatively minor. Other investigators reported a range of changes from large to moderate to small to none, based on the domains of OHRQoL (9).

Several cross-sectional studies investigated the association between OHRQoL and some epidemiological and clinical factors (10). While a wide range of variables have been reported to associate with OHRQoL [which was partially explained based on ethnicity and/or the health system in different countries], Thomson and Malden (11) found in their prospective study no sociodemographic differences in scale scores or changes resulting from DTGA [data were not shown in Thomson and Malden’s article].

The main aim of this study was to assess the mid-term [6 – 9 months] change in children’s OHRQoL after DTGA, paying special attention to several additional issues: [1] the factors associated with pretreatment OHR-QoL scores, [2] the factors associated with changes resulting from DTGA, and [3] a comparison between OHR-QoL pretreatment and post treatment scores of the children who received the DTGA and a cross-matched control group with no complaints related to oral health.

## Material and Methods

A total of 80 children who were 3 to 10 years old participated in this study. All of these children were enrolled from a pediatric dentistry clinic based on their needs of dental treatment under general anesthesia. Patients who were visiting the clinic for regular check-ups and/or prevention purposes [e.g., fluoride application] were excluded. Exclusion criteria comprised also children affected by general disease [e.g., diabetes, disability conditions], denial to participate and failing to attend scheduled consultations in the period of data gathering and preoperative physical examination by an anesthetist. Fifteen patients met the inclusion criteria but excluded from data analyses because they were non-Saudi. All patients’ parents or guardians signed a term of consent to participate in the study. The study protocol was approved by the respective institution’s research committee [FRP/2012/7] and registered by Australian New Zealand Clinical Trials Registry [ACTRN12613000255785]. From January 2012 to May 2013, parents completed the Child Oral Health Quality of Life Questionnaire [COHQoLQ] scale, a form that was specifically designed to assess oral health-related quality of life in this age group. It had been already translated to Arabic and validated for use in Saudi Arabia (12).

The parents completed the questionnaires by themselves with an interviewer remained nearby in case there were any uncertainties. The interviewer readdressed any questions remained unanswered by explaining its meaning to the parent and annotating the answer. Parents who completed the COHQoLQ at baseline completed a similar set of questionnaires after 6-12 months of treatment completion.

The COHQoLQ contains two overall indices on oral health and wellbeing [“How would you rate the health of your child’s teeth, lips, jaws and mouth?” and “How much is your child’s overall wellbeing affected by the condition of his/her teeth, lips, jaws or mouth?”], and 2 instruments known as the Parental-Caregiver Perceptions Questionnaire [P-CPQ] and the Family Impact Scale [FIS]. The P-CPQ consists of 33 specific questions on the following domains of OHRQoL: oral symptoms [6 items], functional limitations [8 items], emotional wellbeing [9 items], and social wellbeing [10 items]. The FIS consists of 16 specific questions on the following domains of OHRQoL: family activity [5 items], parental emotions [4 items] and family conflict [5 items] (5). The P-CPQ was developed to measure the impact of oral health status on the child, whereas the FIS was developed to measure the impact of oral health status on the family. Each question was answered by selecting one of five alternative responses, which assigned scores of 0 to 4, with the higher scores corresponding to a poor OHRQoL. For example, item #3 was the first item in the oral symptom domain and asked about times the child experienced pain in the teeth, lips, jaws or mouth during the last 3 month. The answers for all items ranged from never [score of 0] to once or twice [score of 1] to sometimes [score of 2] to often [score of 3] to every day or almost every day [score of 4]. The postoperative COHQoLQ includes a general question [“Since the operation to fix his/her teeth, is your child’s overall quality of life: much improve, a little improved, the same, a little worse, or much worse”].

Parents answered a supplementary questionnaire on a chief complaint, household socioeconomic status [SES], and other demographic variables [e.g., child’s age and gender, place of residence, respondent’s gender, age and level of education]. Clinical oral examination was carried out by one trained pediatric dentist assisted by a dental assistant for recording the findings related to caries indices [dmft and DMFT] and number of teeth with pulpal involvement and/or dental sepsis.

The cut-off points between children affected by oral health status and those who did not for the total score, scores of the P-CPQ and its domains, and scores of the FIS and its domains, were determined based on the OHRQoL from another sample of children [n = 59] who were cross matched in terms of age, gender, and SES but with no complaints related to oral health. Parents [15 fathers, 44 mothers] of those children completed one set of COHQoLQs.

The sample size was determined based on the difference in total scores of COHRQoL between children having poor OHRQoL compared to control, which was reported in a previous study to be 17 (2). With an alpha of 0.05 and power of 90%, it was determined that 59 patients would be needed from each group. For the pretest-posttest study, it was determined that 74 would be needed based on the least effect size the dental treatment had on children OHRQoL reported by Baghdadi in a previous study (2).

Change scores for the overall OHRQoL, P-CPQ, and FIS were computed by subtracting post treatment scores from pretreatment scores. Change scores were also calculated for each domain or subscale. Paired t-tests were used at *p* < 0.05 to test the statistical significance of the changes. The responsiveness of the questionnaire and the magnitude of changes were determined by dividing the mean of the change scores by the standard deviation of the pretreatment scores. This calculation would give a dimensionless measure of the effect [effect size [ES] statistics of less than 0.2 indicate a small clinically meaningful magnitude of change, 0.2-0.7 a moderate change, and more than 0.7 a large change]. Cross-sectional construct validity was examined by evaluating the association between the means of pretreatment scores and the rating for the question, “How much is your child’s overall wellbeing affected by the condition of his/her teeth, lips, jaws or mouth?” The Kruskal-Wallis test was used to test the statistical significance of the observed association. Internal consistency was assessed using Cronbach’s alpha. A two-step multiple Poisson regression was used to reveal any significant interactions between the various determinants on quality of life before dental treatment. The pretreatment P-CPQ, FIS and domains were dichotomized by the scores of the control group. At the beginning, bivariate analyses were run and explanatory variables presenting a p value 0.20 were included in the final models. The effect of variables on change scores was assessed using multiple linear regression that had changes in P-CPQ domains and FIS domains scores as dependent variables. All tests were run using IBM SPSS Statistics version 22 [IBM Corporation, Somers, NY, USA].

## Results

Eighty parents [29 fathers and 51 mothers] completed the COHQoLQ at baseline and follow-up. The mean age of fathers and mothers were 41.89 [SD 9.25] and 38.87 [SD 7.29], respectively. Eighty patients [41 boys and 39 girls, mean age 6 [SD 2.06]] received the dental treatment, including dental fillings [an average of 6.7 fillings per patient], stainless steel crowns [an average of 0.78 per patient], pulp therapy [an average of 2.77 per patient], and extractions [an average of 1.96 per patient], in addition to preventive measures such as dental sealants and fluoride application. The mean dmft/DMFT was 10.95 [SD 3.08].

**Figure 1:**
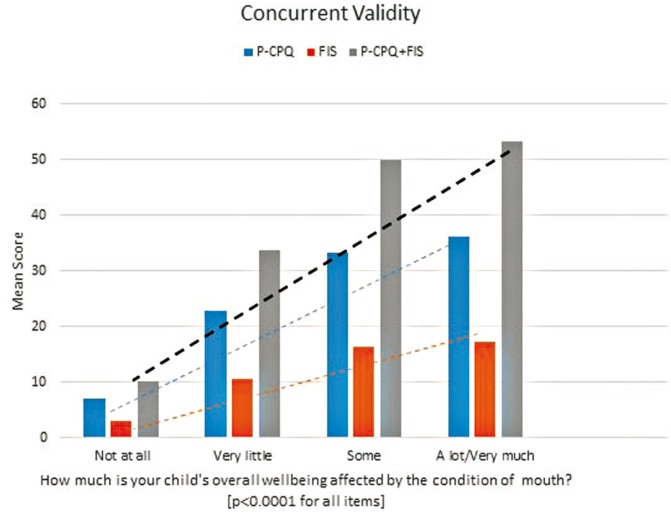
Distribution of responses to the question “How much is the child’s wellbeing affected by his/her mouth,” and the mean of the pretreatment scores for Pretreatment Parental-Caregivers Perceptions Questionnaire (P-CPQ) scale, Family Impact Scale (FIS), and P-CPQ+FIS. Observed gradients support construct validity.

Figure 1 presents the distribution of responses to the question “How much is the child’s wellbeing affected by his/her mouth,” and the mean of the pretreatment scores. Generally, there was a highly significant association between parents’ ratings of children’s oral health status and the P-CPQ, FIS and their domains. In addition, alpha coefficients were exemplary for the P-CPQ [α 0.89], FIS [α 0.81] and the whole COHQoLQ [α 0.91], extensive for all domains [α ranges: 0.71 to 0.83] with the exception of the oral symptom domain, which showed moderate internal consistency [α 0.63]. The parental emotions domain alone showed low internal consistency [α 0.34]; deleting the item “Worried that child will have fewer life opportunities” improved alpha coefficient of the parental emotions domain to 0.59.

**Figure 2:**
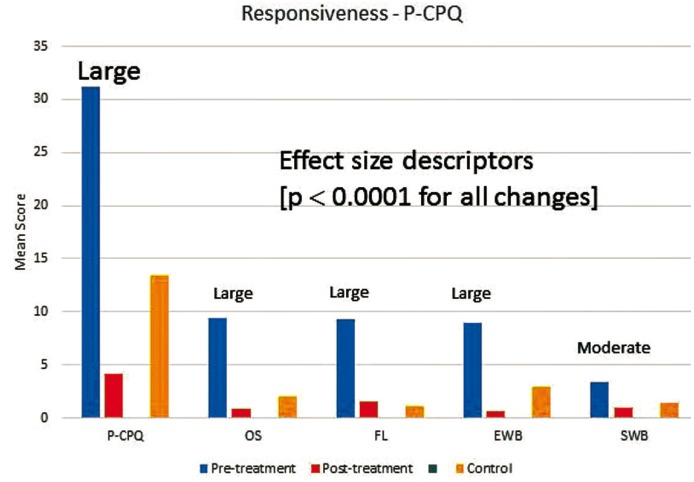
Mean overall and domain (OS oral symptoms, FL functional limitations, EWB emotional wellbeing, SWB social wellbeing) scores in the Parental-Caregivers Perceptions Questionnaire (P-CPQ) at baseline and follow-up, effect sizes, and scores of the control.

**Figure 3:**
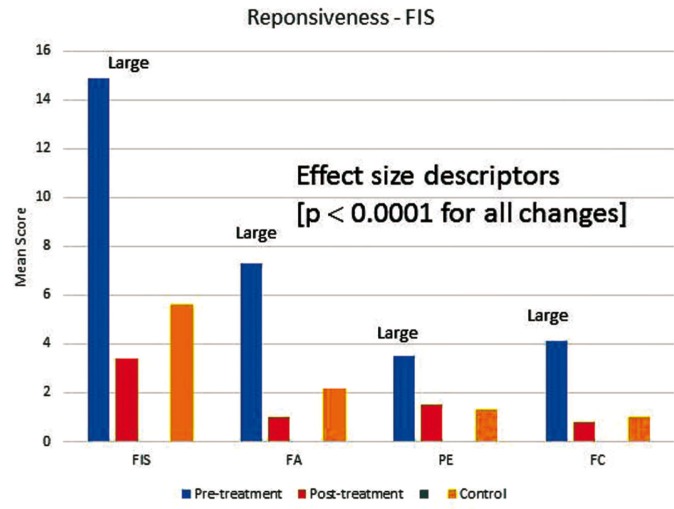
Mean overall and domain (FA family activity, PE parental emotions, FC family conflict) scores in the Family Impact Scale (FIS) at baseline and follow-up, effect sizes, and scores of the control.

Figure 2 and figure 3 present before- and after-treatment scores together with change scores and effect size descriptors for P-CPQ and FIS and their domains. The scores of the control group were also shown in figures. The P-CPQ, FIS and all of their domains showed decreases in mean score associated with dental treatment. The greatest improvement was in the oral symptom domain of the P-CPQ followed by the family activity domain of the FIS. With the exception of the functional limitations and parental emotions domains, all control group scores were higher than post-treatment scores.

 Table 1 presents the association between some epidemiological factors and clinical conditions and scores above the control scores in the P-CPQ and FIS domains. Age presented significant association with all P-CPQ domains, with older children showed worse scores compared to younger children. Other factors presenting significant associations included pain with oral symptoms, functional limitations, and emotional wellbeing and pulpal involvement with oral symptoms.  Table 2 summarizes the results of the multiple regression model in the P-CPQ and FIS domains. After including all statistically significant variables in the Poisson regression model, it was found that the variable age showed a statistically significant association with emotional wellbeing and social wellbeing. In addition, the variable pain showed a statistically significant association with oral symptoms, functional limitations, and emotional wellbeing. For the FIS domains, the variables parent’s gender and pain were statistically significantly associated with the family activities domain and remained the same in the final model, summarized in  Table 2. The variable age showed a statistically significant association with the parental emotions domain, whereas pain was the variable associated with the family conflicts domain ( Table 2).

**Table 1:**
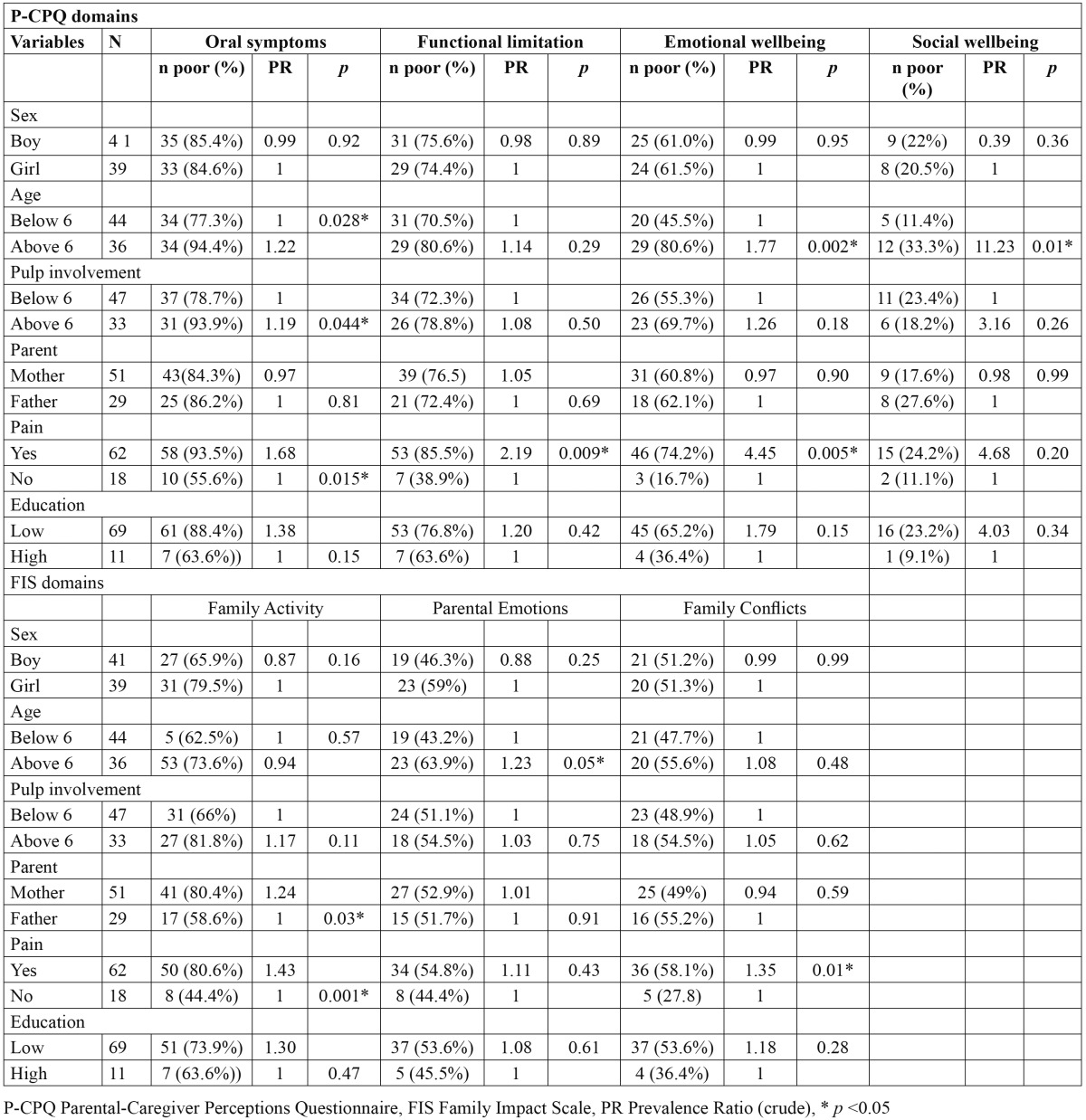
Bivariate analysis of condition variables with OHRQoL in the domains of the P-CPQ and FIS.

**Table 2:**
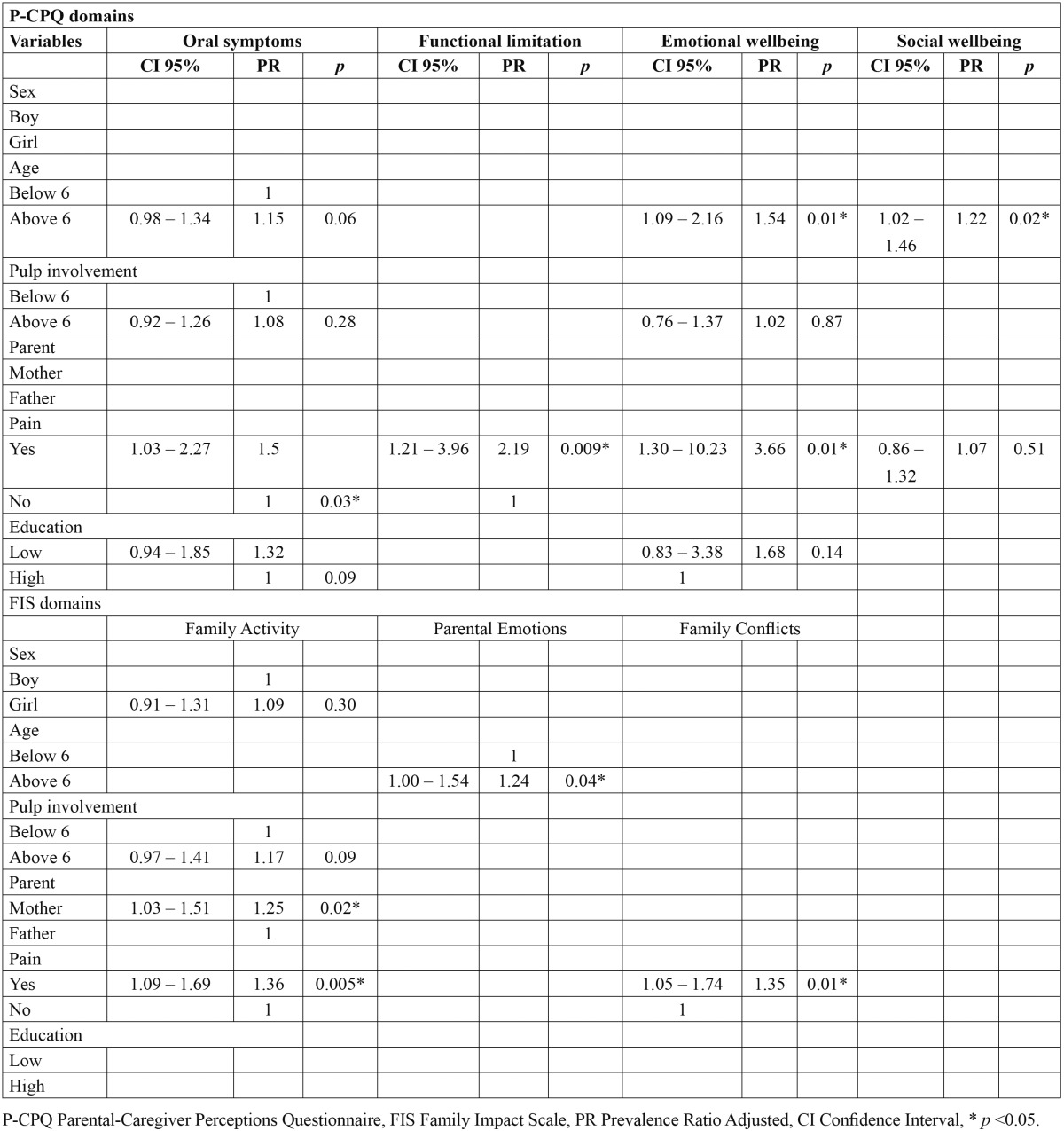
Association between condition variables with OHRQoL in the domains of the P-CPQ and FIS through the Poisson model for multiple regression analysis.

 Table 3 demonstrates the effects of some epidemiological factors on change scores using an ordinary least squares linear regression, with P-CPQ, FIS and their domains change scores as separate dependent continuous variables. The epidemiological variables tested were those statistically significantly associated with pretreatment scores. From all factors examined, pain was statistically associated with change scores in all scales and subscales with the exception of social wellbeing, which was associated with age. Age was also the variable associated with change scores in P-CPQ and oral symptoms domain.

**Table 3:**
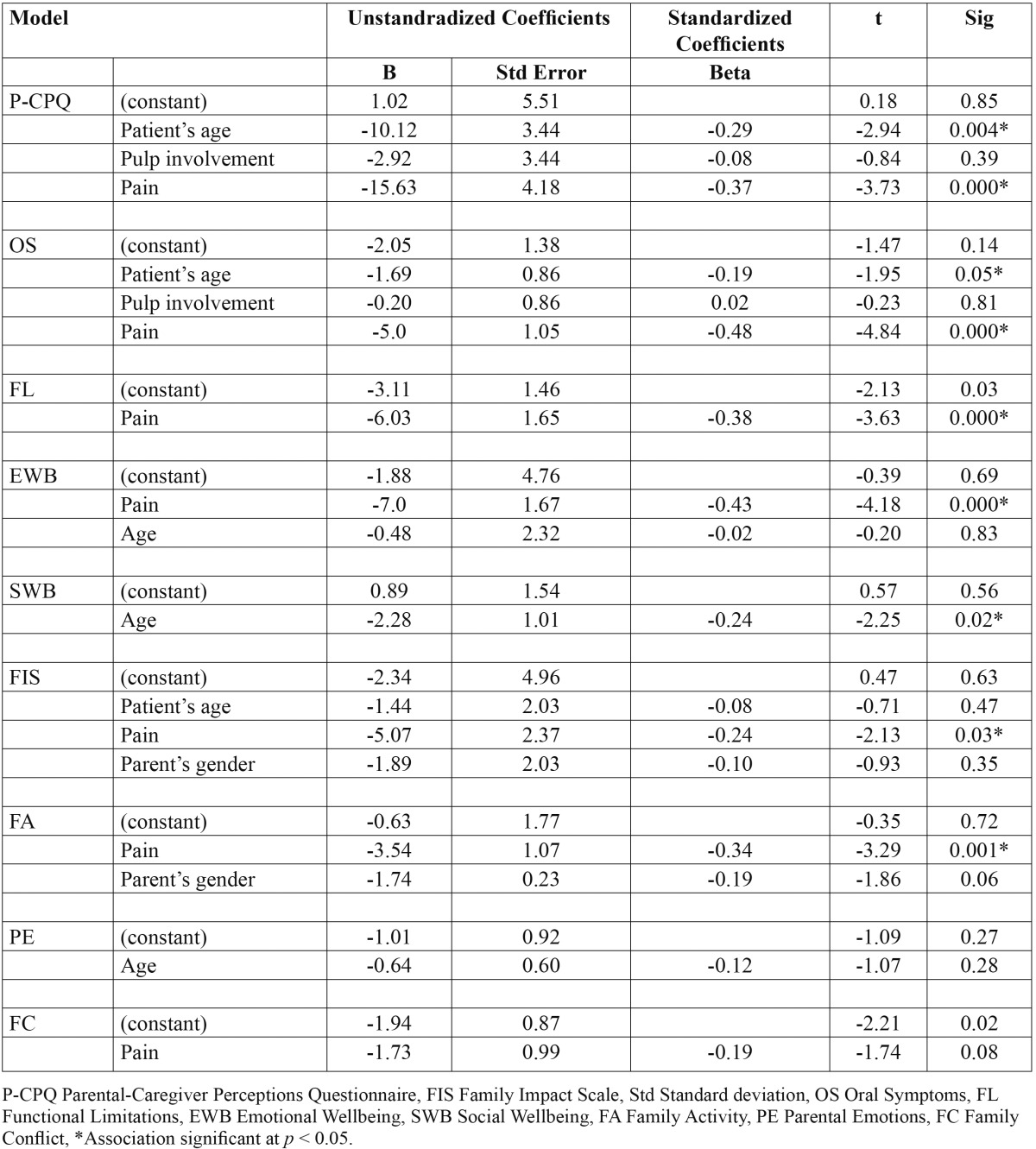
Multiple linear regression of epidemiological and clinical variables effects on change scores of P-CPQ, FIS, and their domains.

## Discussion

The results of the current study documented the mid-term positive impact of DTGA on children’s OHRQoL. The effect sizes of change were large for the P-CPQ, FIS, and all their domains with the exception of social wellbeing, which showed a moderate effect. More importantly, the post treatment OHRQoL scores were, in general, lower than those of the control group, indicating that the effect found is caused by the treatment. The minimal clinically important difference [MCID] is the statistical model aims to define the smallest change in a treatment outcome identified important by a patient [or proxy, such as health caregiver or parent] (5). In the current study, answering the general postoperative question about children’s improvement as a result of the operation, 73 [91.3%] parents reported that children much improved, 4 [5%] parents reported a little improvement, and 3 [3.8%] reported the same. A similar distribution was demonstrated in a shorter follow-up study [4 – 8 weeks] (2). The skewness of the answers precluded calculation of the MCID using the anchor-based method. However, effect size [which is based on SDs] offers a reliable measure to demonstrate the responsiveness of the scale and the magnitude of changes (5).

The results of the current study concerning the scale’s responsiveness and the magnitude of changes are in agreement with a similar previous study on Saudi children, which documented considerable change scores after 4 -8 weeks of DTGA using both short-form versions (2) and full-form versions of the P-CPQ and FIS [Baghdadi & Muhajarine, under preparation]. Similar, but short-term, positive results were reported by others using full-form versions of the scales (5,11). In addition to responsiveness, internal consistency [assessed by Cronbach’s alpha] is another important feature of quality of life questionnaires. With the exception of parental emotions domain of the FIS [which showed low internal reliability], the internal consistency reliability was substantial to excellent for the P-CPQ, FIS and all their domains, as well as for the whole scale [P-CPQ plus FIS]. Thomson and Malden (11) found that the parental emotions domain had low internal reliability, which was related to “Worried that child will have fewer life opportunities” and “Felt uncomfortable in public places with your child.” Klaassen *et al.* (6) found a similar problem with the family conflicts domain, which was related to the financial impact the condition has on the family, considering that the healthcare system in the Netherlands covers the costs of DTGA. This indicates the importance of modifying some items before a scale developed in a certain region/population/language can be used in another region/population/language. A similar conclusion was recently reported by Albites *et al.* (13) who examined the psychometric properties of the P-CPQ in the Peruvian Spanish language.

Cross-sectional studies have shown that clinical and socio-environmental factors had different impacts on children’s OHRQoL in general as well as on each domain (10,14). Considering these conditions is important in planning strategies for the oral health of children. In the current study, child’s age, pain, and number of teeth with pulpal involvement/sepsis were associated with pretreatment scores, whereas child’s gender, parent’s gender, and parent’s level of education had not shown such an association. Those variables [age, pain, pulpal involvement] were also strongly associated with change scores. This is consistent with the fact that dental pain was found to be the main reason for visiting the dentist among Saudi schoolchildren (3). A 4-months study on Filipino children found that extraction of severely decayed and pulpally-involved teeth was associated with weight gain concomitant with an improvement in children’s eating and sleeping (15). Age, in turn, plays an important factor in chronic diseases, such as dental caries, because severe manifestations of a dental decay require time to develop and have a discernable impact on an individual. A recent systematic review of the impact of parental socioeconomic status and home environment characteristics on children’s OHRQoL found that most of the studies were conducted on Brazilian children, were published in 2012-2013, and were of low or moderate quality because they are not longitudinal (10). Definitive conclusions from the studies were not possible because of the differences in the study population, characteristics considered, and methods used. This area requires further prospective research. Long-term effects of DTGA on children’s OHRQoL need documentation. In conclusion, this study demonstrated a mid-term large improvement in children’s oral health-related quality of life as a result of comprehensive dental treatment completed under general anesthesia, as reported by children’s parents. It also demonstrated a strong association between children’s OHRQoL and changes resulting from dental treatment and variables like patient’s age, pain, and number of pulpally-involved teeth. Those variables can be used as predictors for both pretreatment scores and change scores following treatment.
